# Potential Mechanisms of an Antiadenomyosis Chinese Herbal Formula Shaoyao-Gancao Decoction in Primary Cell Culture Model

**DOI:** 10.1155/2014/982913

**Published:** 2014-11-10

**Authors:** Yong-Ge Guan, Jin-Bin Liao, Kun-Yin Li, Yu-Cui Li, Yang Song, Jing Ling, Zi-Ren Su

**Affiliations:** ^1^First Affiliated Hospital, Guangzhou University of Chinese Medicine, Guangzhou 510405, China; ^2^School of Chinese Materia Medica, Guangzhou University of Chinese Medicine, Guangzhou 510006, China; ^3^Guangdong Second Province Hospital of Traditional Chinese Medicine, Guangzhou 510095, China; ^4^School of Nursing, Guangzhou University of Chinese Medicine, Guangzhou 510405, China

## Abstract

*Background*. Shaoyao-Gancao Decoction (SGD), a well-known traditional Chinese medicine prescription, has been widely used to treat adenomyosis, dysmenorrhea, abdominal pain, and inflammation in Asia. However, the mechanism underlying the effectiveness of SGD in the treatment of adenomyosis still remains elusive. The present study aimed to investigate the bioactivity of SGD and its underlying molecular mechanisms using cultured human adenomyosis-derived cells. *Methods*. Human adenomyosis-derived cells were treated with SGD and its major constituents (paeoniflorin and liquiritin) *in vitro*. Effects of SGD, paeoniflorin, and liquiritin on cell proliferation and apoptosis were examined by MTT assay and flow cytometry analyses. The effects of SGD, paeoniflorin, and liquiritin on the production of PGE_2_ and PGF_2*α*_ were assayed using ELISA. ER-*α* and OTR mRNA expression levels were also evaluated by real-time qRT-PCR. *Results*. SGD, paeoniflorin, and liquiritin inhibited proliferation and induced apoptosis of human adenomyosis-derived cells in a dose-dependent manner. SGD and paeoniflorin significantly reduced the PGE_2_ and PGF_2*α*_ production. Furthermore, they remarkably decreased the mRNA levels of ER-*α* and OTR. *Conclusions*. The results of this study provide possible mechanisms for the bioactivity of SGD for treating adenomyosis and contribute to the ethnopharmacological knowledge about this prescription.

## 1. Introduction

Adenomyosis, also known as endometriosis interna, is a common gynecological disorder defined as the benign invasion of endometrial glands and stroma into the myometrium [1]. The reported prevalence of adenomyosis varies from 5% to 70% [2]. Adenomyosis is not a fatal disease, but it does have a tremendous impact on the quality of a woman's life, causing many health problems, such as dysmenorrhea, menorrhagia, metrorrhagia, early pregnancy-stage miscarriages, or even subfertility [3]. Treatment of adenomyosis has been a challenge, with hysterectomy being the treatment of choice for severe and symptomatic adenomyosis, yet hysterectomy can be quite traumatic for women who still wish to have a family [4]. Even though adenomyosis is recognized as hormone sensitive, progestogenic agents are ineffective [5]. Other therapeutic options include gonadotrophin-releasing hormone (GnRH) agonists that could induce suppression of adenomyosis. However, their use is confined by short duration and easy recurrence after discontinuation of therapy [6, 7]. Moreover, uterine artery embolization (UAE) has been investigated as a possible therapy for adenomyosis, but the effectiveness of UAE in the treatment of adenomyosis is still limited [8, 9]. Thus, novel therapeutic strategies are urgently needed to improve the clinical management of patients with adenomyosis.

Shaoyao-Gancao Decoction (SGD, Shakuyaku-Kanzo-to in Japanese), a well-known traditional Chinese medicine prescription, was sourced from the Chinese Medical Classics text—*Shanghan lun* in 210 CE. The herbal prescription, which is made up of two herbs (*Paeoniae Radix* and* Glycyrrhizae Radix*, “Shaoyao” and “Gancao” in Chinese, resp.), is commonly used in the treatment of gynecological disorders in China, including dysmenorrheal [10, 11], menorrhagia [12], and infertility [10]. Clinically, SGD has been used very efficaciously and widely to treat adenomyosis in women in traditional Chinese medical practice, since it has the advantage of noninvasive and less/no side effects [13, 14]. Similar herbal prescription has also been used in Japan and other Oriental countries [15]. In addition, modern pharmacological studies have demonstrated that SGD possesses analgesic [16] and anti-inflammatory [17] effects. Moreover, SGD treatment resulted in a significantly low incidence of adenomyosis in an experimental animal model [18].

However, little is known about the potential mechanisms of the antiadenomyosis effect of SGD. In this study, we examined the effects of SGD and its major constituents (paeoniflorin and liquiritin) on the proliferation and apoptosis of human adenomyosis-derived cells. In addition, their effects on prostaglandin E_2_ (PGE_2_) and prostaglandin F_2*α*_ (PGF_2*α*_) production and estrogen receptor-*α* (ER-*α*) mRNA and oxytocin receptor (OTR) mRNA expression were also investigated.

## 2. Materials and Methods

### 2.1. Materials

Acetonitrile (Merk, Darmstadt, Germany); Paeoniflorin and liquiritin were obtained from The National Institute for the Control of Pharmaceutical and Biological Products (Beijing, China); 3-(4, 5-dimethylthiazol-2-yl)-2, 5-diphenyltetrazolium bromide (MTT), Apoptosis Detection Kit (Catalog no. V13241), sodium dodecyl (lauryl) sulphate-hydrochloride (SDS-HCl), and Trizol were purchased from Invitrogen; Dulbecco's modified Eagle's medium (DMEM), Collagenase I, 0.25% trypsin, fetal bovine serum (FBS), and phosphate buffered saline (PBS) were obtained from Gibco; M-MLV RTase cDNA Synthesis Kit and SYBR Premix Ex Taq were obtained from Takara (Dalian, China); PGE_2_ enzyme-linked immunosorbent assay (ELISA) kit was obtained from R&D Systems (Minneapolis, USA); PGF_2*α*_ ELISA kit was purchased from ENZO Life Sciences.

### 2.2. Single Herb and SGD Preparation

*Paeoniae Radix* and* Glycyrrhizae Radix* were purchased from Caizhilin Materials Company of Guangzhou and authenticated by professor Zi-Ren Su in the School of Pharmacy of Guangzhou University of Chinese Medicine.

According to the preparation method of SGD by Shen et al. with slight modifications [19], extract solutions of two single herbs and SGD were prepared in the following procedure. Two single herbs and SGD were macerated for 30 min with eight times (v/w) distilled water. The medical solutions were heated to boiling and then decocted for 40 min and the filtrates were collected. The residues were decocted again for 30 min with eight times (v/w) distilled water, then collected filtrates again, and were mixed with previous collected filtrates, condensed under reduced pressure at 80°C to obtain a concentration equivalent to 1 g/mL crude herb materials and stored at −20°C.

### 2.3. Ultra-Fast Liquid Chromatography (UFLC) Analysis

Ultra-fast liquid chromatography (UFLC) profiles of two single herbs and SGD were performed as the following procedures: extract solutions of* Paeoniae Radix* (0.2 mL),* Glycyrrhizae Radix* (0.08 mL), and SGD (0.28 mL) with a concentration equivalent to 1 g/mL crude herb materials were dried and then extracted with methanol-water (10 mL, 50 : 50, v/v) under ultrasonication for 30 min. The solutions were filtered and then determined for UFLC analysis.

UFLC equipment was controlled with LC-20AD pump (Shimadzu Co., Kyoto, Japan) using a YMC-UltraHT Pro C_18_ column (50 mm × 2.0 mm ID, 2 *μ*m; YMC, Inc.). The elution gradient was carried out with binary solvent system consisting of 0.5% acetic acid in H_2_O (solvent A) and MeCN (solvent B). A linear gradient profile with the following proportions (v/v) of solvent B was applied: gradient profile 0 to 10 min and 5% of B, 10 to 20 min and 5% to 15% of B, 20 to 30 min and 15% to 30% of B, 30 to 40 min and 30% to 50% of B, and 40 to 45 min and 50% to 70% of B. The flow rate was controlled with LC 20AD at 0.4 mL/min, injection volume 5 *μ*L, and the column temperature was maintained at 28°C. The effluents from the column were detected at absorbencies ranging from 200 to 400 nm using a photodiode-array detector. The three-dimensional data collection and processing for peak analysis were accomplished with CLASS-LC10 System Analysis Software (Shimadzu Co., Kyoto, Japan).

### 2.4. Tissue Preparation

Adenomyosis specimens were obtained at the time of laparoscopy or laparotomy performed for adenomyosis treatment from eight Chinese women aged 20–45 years. All patients had regular menstrual cycles. No patients were undergoing hormone therapy prior to surgery. All biopsy specimens were collected at the First Affiliated Hospital of Guangzhou University of Traditional Chinese Medicine between November 2009 and August 2010 with the permission of the local ethics committee, and written informed consent was obtained from each patient.

### 2.5. Cell Isolation and Culture

The biopsy specimens were immediately placed in ice-cold DMEM for transport to the laboratory. The tissues were then dissociated as previously described with some modifications [20]. The biopsy specimens were washed once with PBS and twice with DMEM. The biopsy specimens were then minced into small pieces and incubated at 37°C for 5 to 6 hours in Hank's balanced salt solution supplemented with 0.1% type I collagenase. DMEM containing 10% FBS was added. The cell suspensions were filtered through a 200 mesh stainless steel screen, and the filtrates were centrifuged (1000 rpm, 8 min, 3 times). Cells were suspended at a concentration of 5 × 10^5^ cell/mL in DMEM supplemented with 10% FBS, then transferred to a tissue culture dish, and incubated at 37°C. In order to accumulate enough cells for further study, cultured cells of individual patients were pooled and cryopreserved. The lower passage number (three to six) of cells was used for experiments to avoid changes in phenotype and gene expression.

### 2.6. Cell Survival Assay

Isolated cells were first placed in 96-well microtiter plates at a density of 5 × 10^4^ cells per well and cultivated in 100 *μ*L of the DMEM containing 10% FBS. After a 3-day preincubation period, culture media were replaced with 100 *μ*L DMEM and then incubated at 37°C for 24 h. The supernatants were removed, and 100 *μ*L of fresh DMEM containing various concentrations of SGD (0.1–500 mg/mL), paeoniflorin (0.0001–10 mg/mL), liquiritin (0.0001–10 mg/mL), and mifepristone (0.005–100 *μ*M as positive control) was added. Cells were incubated at 37°C for 24 h; then 10 *μ*L MTT (5 mg/mL) was added, and the cells were incubated at 37°C for 4 h. After the medium and MTT were removed, 100 *μ*L of SDS-HCL was added to each well and then placed on a plate shaker for 10 min at room temperature. For each well, absorbance at 570 nm was measured using a microtiter plate ELISA reader. The cell survival rate was calculated as follows: percent survival =  (mean experimental absorbance/mean control absorbance) × 100.

### 2.7. Flow Cytometric Analysis of Cell Apoptosis

The flow cytometric analysis of annexin V-FITC and propidium iodide (PI) stained cells was performed using the Apoptosis Detection Kit [21]. Annexin V binds to extracellular phosphatidylserine, a marker for both apoptotic cells and necrotic cells. PI can enter only cells in which the integrity of the membrane has been compromised, which can constitute necrotic or late apoptotic cells. Therefore, healthy cells are doubly negative to annexin V and PI. Cells stained positively only for annexin V are early apoptotic. Cells that are doubly positive for annexin V and PI are considered to be in late apoptosis [22]. According to the manufacturer's protocol, after treatments with SGD (10 and 20 mg/mL), paeoniflorin (0.25, and 0.5 mg/mL), liquiritin (0.25 and 0.5 mg/mL), and mifepristone (10 *μ*M as positive control) for 24 hours, cells were collected and washed with PBS. Cells were harvested by incubating with 0.25% trypsin for 3–6 min, and then DMEM containing 10% FBS was added. Cells were centrifuged at 1000 rpm for 5 min and then washed 3 times with PBS, followed by being resuspended in 500 *μ*L of binding buffer containing 5 *μ*L annexin V-FITC and 5 *μ*L PI and then incubated for 5 min in the dark at room temperature. Following this, the cells were analyzed by flow cytometry. The percentage of cells undergoing apoptosis was determined by three independent experiments.

### 2.8. Measurement of PGE_2_ and PGF_2*α*_ Production

Human adenomyosis-derived cells were pretreated with SGD (10 and 20 mg/mL), paeoniflorin (0.25 and 0.5 mg/mL), and liquiritin (0.25 and 0.5 mg/mL) for 24 hours. The concentrations of PGE_2_ and PGF_2*α*_ in the culture supernatant were determined by ELISA kits according to the manufacturer's protocol.

### 2.9. Real-Time Quantitative Reverse Transcription-Polymerase Chain Reaction (qRT-PCR) Analysis

The mRNA levels of ER-*α* and OTR were determined by qRT-PCR analysis. Cells preincubated with or without SGD (10 and 20 mg/mL), paeoniflorin (0.25 and 0.5 mg/mL), and liquiritin (0.25 and 0.5 mg/mL) for 24 hours, and then total RNA was isolated using Trizol Reagent according to the manufacturer's protocol. Total RNA was reverse-transcribed using a cDNA synthesis kit. qRT-PCR was performed on the Thermal Cycler Dice Real Time System using SYBR Premix Ex Taq. The forward and reverse primers were 5′-GTCTCGTCTGGCGCTCCAT-3′ and 5′-CCCTGGTTCCTGTCCAAGAG-3′ for ER*α* mRNA; 5′-AGGAAGCCTCGGCCTTCAT-3′ and 5′-GAGGTGGCCCGTGAACAG-3′ for OTR mRNA; 5′-GCATGGGTCAGAAGGATTCCT-3′ and 5′-TCGTCCCAGTTGGTGACGAT-3′ for *β*-actin mRNA. The relative expression of ER-*α* and OTR mRNA was normalized to the amount of *β*-actin mRNA levels.

### 2.10. Statistical Analysis of Data

For all groups data are presented as the mean plus or minus standard deviation (SD). Statistical significance within a parameter was evaluated by one-way analysis of variance (ANOVA) using the SPSS. Value of *P* < 0.05 was considered statistically significant.

## 3. Results

### 3.1. Chemical Profiles of SGD

**Figure 1:**
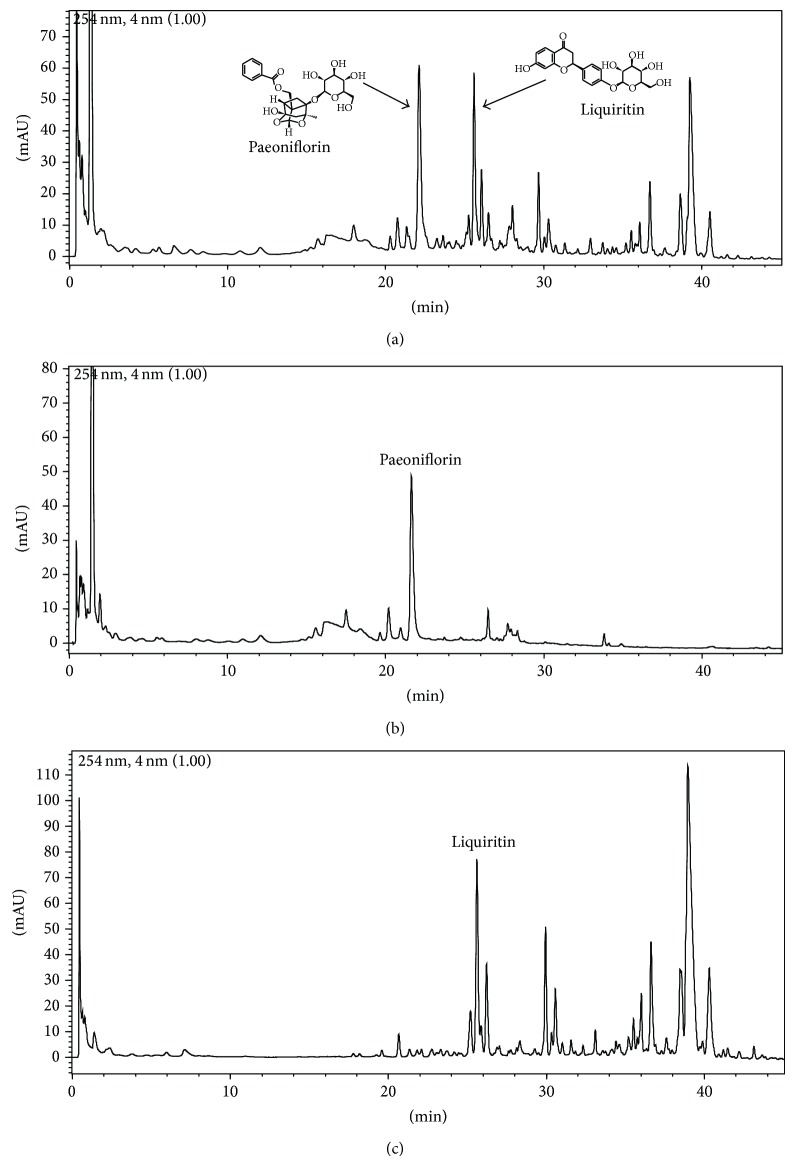
UFLC pattern of SGD and single herb. (a) SGD, (b) Bai Shao (*Paeonia lactiflora* Pall., Radix), and (c) Gan Cao (*Glycyrrhiza uralensis* Fisch., Radix et Rhizoma); each herb was extracted with water as described in the section of methods. The dried powder of each herb was extracted with methanol-water (10 mL, 50 : 50, v/v) under ultrasonication for 30 min. The solutions were filtered and then determined for UFLC analysis.

UFLC profiles of two single herbs and SGD were shown in Figure 1. In order to control the quality of SGD, we also detected the two active compositions of five batches of SGD by UFLC with standard compositions. The results indicated all the five batches contained two active compounds. The RSD of the relative peak areas were between 0.32% and 2.57%.

### 3.2. Cell Morphology

Inverted microscope was selected for the identification of human adenomyosis-derived cells. Examination of these cells in culture showed monolayers of cells, displaying a fibroblast-like, stellate, or spindle-like morphology, as reported by Suzuki-Kakisaka et al. [23].

### 3.3. Cytotoxic Effects of SGD and Its Constituents on Human Adenomyosis-Derived Cells

**Figure 2:**
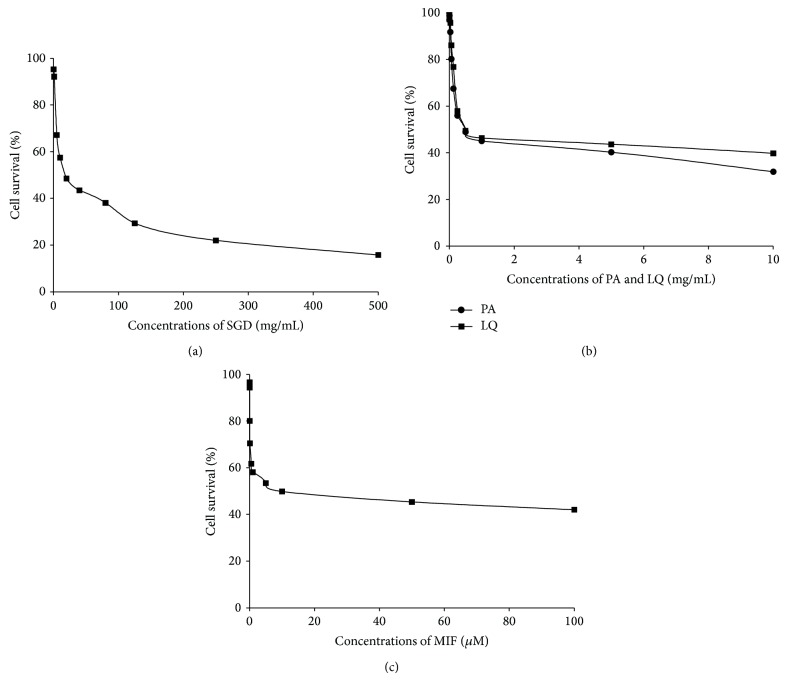
Cytotoxic effects of SGD and its constituents on human adenomyosis-derived cells. After treatments with (a) SGD, (b) paeoniflorin (PA) and liquiritin (LQ), and (c) mifepristone (MIF), the survival of human adenomyosis-derived cells was measured by the MTT assay. The results displayed that SGD, paeoniflorin, liquiritin, and mifepristone significantly inhibited the survival of human adenomyosis-derived cells. The IC_50_ values were 25.06 mg/mL, 1.03 mg/mL, 1.62 mg/mL, and 10.61 *μ*M, respectively. Representative graphs are shown.

The inhibitory effect of SGD, paeoniflorin, and liquiritin on the growth of human adenomyosis-derived cells was evaluated using MTT assay. The effects on human adenomyosis-derived cells are shown in Figure 2, where the cells were treated with different concentrations of SGD (0.1–500 mg/mL), paeoniflorin (0.0001–10 mg/mL), liquiritin (0.0001–10 mg/mL), and mifepristone (0.005–100 *μ*M) for 24 h. The results showed that SGD, paeoniflorin, liquiritin, and mifepristone inhibited the survival of human adenomyosis-derived cells in a dose-dependent manner. The IC_50_ value of SGD, paeoniflorin, liquiritin, and mifepristone was 25.06 mg/mL, 1.03 mg/mL, 1.62 mg/mL, and 10.61 *μ*M, respectively.

### 3.4. SGD and Its Constituents Induce Apoptosis in Human Adenomyosis-Derived Cells

**Figure 3:**
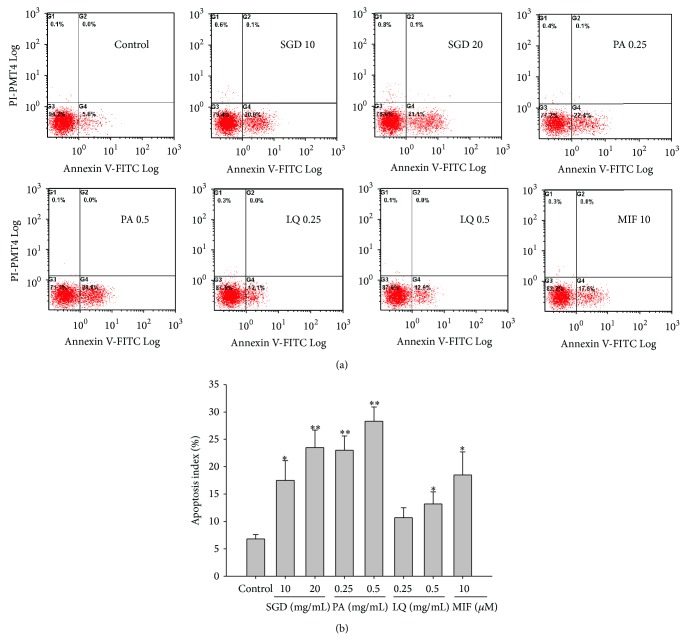
SGD and its constituents, paeoniflorin (PA) and liquiritin (LQ), treatment results in apoptosis of human adenomyosis-derived cells. Apoptotic cells were assessed by annexin V-FITC and PI staining as described in Section 2. Mifepristone (MIF) was used as positive control. The data were expressed as mean ± SD. Three independent experiments were performed. ^*^ *P* < 0.05 and ^**^ *P* < 0.01 indicated the groups with significant difference when compared to the control group.

Simultaneous staining with annexin V-FITC and PI distinguished between healthy, early apoptotic, late apoptotic, and necrotic cells [24]. After 24 h of treatment with or without different concentrations of SGD (10 and 20 mg/mL), paeoniflorin (0.25 and 0.5 mg/mL), liquiritin (0.25 and 0.5 mg/mL), and mifepristone (10 *μ*M), human adenomyosis-derived cells were analyzed by flow cytometry.  Figure 3 shows the percentages of apoptotic cells that were undergoing early apoptosis and late apoptosis. The apoptosis rate in human adenomyosis-derived cells was 6.8 ± 0.8% when the cells were not treated with SGD, paeoniflorin, liquiritin, and mifepristone (control group). However, the percentage of apoptotic human adenomyosis-derived cells was increased to 17.5 ± 3.6% (*P* < 0.05), 23.5 ± 3.2% (*P* < 0.01), 23.0 ± 2.6% (*P* < 0.01), 28.3 ± 2.6% (*P* < 0.01), 10.7 ± 1.8%, 13.2 ± 2.2% (*P* < 0.05), and 18.5 ± 4.2% (*P* < 0.05) in the presence of SGD (10 and 20 mg/mL), paeoniflorin (0.25 and 0.5 mg/mL), liquiritin (0.25 and 0.5 mg/mL), and mifepristone (10 *μ*M), respectively. These results demonstrated that SGD (10 and 20 mg/mL), paeoniflorin (0.25 and 0.5 mg/mL), liquiritin (0.5 mg/mL), and mifepristone (10 *μ*M) could significantly induce the apoptosis of human adenomyosis-derived cells.

### 3.5. Detection of PGE_2_ and PGF_2*α*_ in the Supernatants

**Figure 4:**
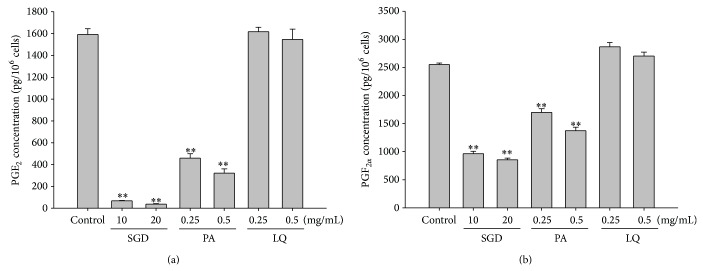
Effects of SGD and its constituents on the production of PGE_2_ and PGF_2*α*_ in human adenomyosis-derived cells. Cells were treated with SGD, paeoniflorin (PA), and liquiritin (LQ). Panels (a) and (b) show results of PGE_2_ and PGF_2*α*_, respectively. Both PGE_2_ and PGF_2*α*_ production in the supernatant were significantly decreased by the treatment of SGD and PA. Data represented the mean ± SD of three independent experiments. Significant lower PGE_2_ and PGF_2*α*_ compared to the control group (^*^ *P* < 0.05 and ^**^ *P* < 0.01).

To investigate the potential mechanism underlying the effectiveness of SGD in the treatment of adenomyosis-associated dysmenorrhea, the levels of two well-established pain mediators, namely, PGE_2_ and PGF_2*α*_, were examined. As shown in Figure 4, SGD (10 and 20 mg/mL) and paeoniflorin (0.25 and 0.5 mg/mL) dose-dependently and significantly decreased the production of PGE_2_ (69.00 ± 2.53 pg/10^6^ cells, *P* < 0.01; 37.17 ± 4.58 pg/10^6^ cells, *P* < 0.01; 458.81 ± 40.81 pg/10^6^ cells, *P* < 0.01; 322.16 ± 38.41 pg/10^6^ cells, *P* < 0.01) and PGF_2*α*_ (966.07 ± 40.25 pg/10^6^ cells, *P* < 0.01; 856.70 ± 26.94 pg/10^6^ cells, *P* < 0.01; 1699.93 ± 64.97 pg/10^6^ cells, *P* < 0.01; 1372.04 ± 62.15 pg/10^6^ cells, *P* < 0.01), while PGE_2_ and PGF_2*α*_ in control group were 1592.63 ± 51.35 and 2553.58 ± 25.69 pg/10^6^ cells, respectively. These results suggested that the decrease in production of PGE_2_ and PGF_2*α*_ might be a potential mechanism in relieving adenomyosis-associated dysmenorrhea using SGD. Meanwhile, the activity of SGD in decreasing PGE_2_ and PGF_2*α*_ production might be partly ascribable to paeoniflorin.

### 3.6. ER-*α* mRNA and OTR mRNA Expression in Human Adenomyosis-Derived Cells

**Figure 5:**
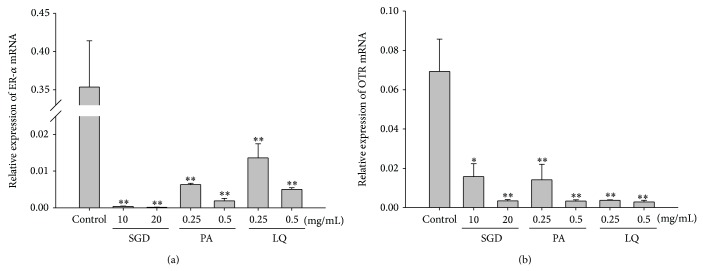
Effects of SGD and its constituents on the expression of (a) ER-*α* mRNA and (b) OTR mRNA in human adenomyosis-derived cells. The mRNA levels in human adenomyosis-derived cells from control and SGD, paeoniflorin (PA), and liquiritin (LQ) treatment groups were determined by qRT-PCR. Data represented the mean ± SD of three independent experiments. ^*^ *P* < 0.05 and ^**^ *P* < 0.01, versus controls.

The effects of SGD, paeoniflorin, and liquiritin on the expression of ER-*α* mRNA and OTR mRNA were investigated by qRT-PCR. As shown in Figure 5, treatments with SGD (10 and 20 mg/mL), paeoniflorin (0.25 and 0.5 mg/mL), and liquiritin (0.25 and 0.5 mg/mL) significantly decreased the mRNA levels of ER-*α* (0.000390 ± 0.000099, *P* < 0.01; 0.000136 ± 0.000051, *P* < 0.01; 0.006342 ± 0.000343, *P* < 0.01; 0.001910 ± 0.000704, *P* < 0.01; 0.013642 ± 0.003817, *P* < 0.01; 0.005028 ± 0.000456, *P* < 0.01), in comparison to control group (0.353722 ± 0.060531). In addition, SGD (10 and 20 mg/mL), paeoniflorin (0.25 and 0.5 mg/mL), and liquiritin (0.25 and 0.5 mg/mL) obviously decreased the mRNA levels of OTR (0.0157 ± 0.0066, *P* < 0.05; 0.0034 ± 0.0007, *P* < 0.01; 0.0141 ± 0.0079, *P* < 0.01; 0.0033 ± 0.0007, *P* < 0.01; 0.0037 ± 0.0003, *P* < 0.01; 0.0028 ± 0.0009, *P* < 0.01) in a dose-dependent manner, in comparison to control group (0.0693 ± 0.0164).

## 4. Discussion

Traditional Chinese medicines have attracted interest and acceptance in many countries with the merits of few side effects, affordability, and local availability [25]. However, there are few of the traditional Chinese medicines that have been illustrated clearly how it works. SGD, a famous traditional Chinese prescription, is widely used in clinic to relieve many types of pain, especially dysmenorrhea in women with endometriosis and adenomyosis [11, 14]. Therefore, the potential mechanism of the antiadenomyosis effects of this traditional prescription is worth investigation. In this study, we provide evidence that SGD and its active ingredients, paeoniflorin and liquiritin, have a beneficial effect in the treatment of adenomyosis.

Cell proliferation has been shown to have multiple effects in embryonic development and pattern formation, including cell growth, morphogenesis, and gene expression [26]. However, abnormal cell proliferation may cause cancer, and adenomyosis has been found to be associated with abnormal endometrial proliferation [27, 28]. The present study showed that SGD had a significant inhibitory effect on the survival of human adenomyosis-derived cells in a dose-dependent manner. Furthermore, increased cellular proliferation has been shown to contribute to the pathogenesis of abnormal uterine bleeding associated with adenomyosis [29]. These results showed that SGD could reduce proliferation of human adenomyosis-derived cells, which indicated the treatment of SGD could prevent the formation of new adenomyotic lesions.

Apoptosis, a process of programmed cell death, is essential for embryonic development, proper development and functioning of the immune system, and the maintenance of tissue homeostasis [30]. Accumulated evidence suggests that apoptosis helps to maintain cellular homeostasis during the menstrual cycle by eliminating senescent cells from the functional layer of the uterine endometrium during the late secretory and menstrual phase of the cycle [31]. However, inappropriate apoptosis (either too little or too much) may contribute to the pathology of many human diseases, including neurodegenerative diseases, ischemic damage, autoimmune disorders, and cancer [32]. It has been reported that a decrease in apoptosis led to ectopic survival and implantation of endometrial cells and development of adenomyosis [33]. These studies indicate that apoptosis-inducing agents can be a potential therapeutic strategy for the treatment of adenomyosis [34]. In the present study, quantification of apoptosis via annexin V and PI staining was performed, and the results showed that SGD dose-dependently enhanced apoptosis in human adenomyosis-derived cells. These results suggested that the prevention and treatment of adenomyosis using SGD might be partially attributed to increased apoptosis in adenomyotic cell.

Dysmenorrhea, defined as painful menstruation involving low abdominal pains, is a major clinical symptom of adenomyosis [35, 36]. Prostaglandins (PGs), especially PGE_2_ and PGF_2*α*_, are bioactive lipids derived from arachidonic acid and play an important role in the etiology of dysmenorrhoea [37, 38]. In previous studies, it has been reported that the concentrations of PGE_2_ and PGF_2*α*_ were higher in the menstrual fluid of women with dysmenorrhoea than in women with painless periods [37]. The increased production of PGE_2_ and PGF_2*α*_ could upregulate cyclooxygenase-2 (COX-2), which is an essential enzyme for the synthesis of PGE_2_ and PGF_2*α*_, causing dysmenorrhea in adenomyosis [39, 40]. Therefore, suppression of PGE_2_ and PGF_2*α*_ synthesis has become the main treatment of dysmenorrhoea [41]. In the present study, an assay on the levels of PGE_2_ and PGF_2*α*_ was performed. Results showed that treatment with SGD dose-dependently and significantly decreased the production of PGE_2_ and PGF_2*α*_. A similar result has been reported by Imai et al. that incubation of human endometrial cells with SGD induced a significant decrease in PG levels [14]. In addition, Shibata et al. also reported that concentrations of PGE_2_ and PGF_2*α*_ in the culture medium of human myometrial biopsies were significantly decreased by the addition of SGD [42]. The above findings suggested that SGD might alleviate adenomyosis-associated dysmenorrhea by decreasing PGE_2_ and PGF_2*α*_ production.

Adenomyosis is most frequent in women of reproductive age and regress after menopause, which is well regarded as an estrogen-dependent condition [43]. The estrogen and its classical estrogen receptors (ERs), ER-*α* and ER-*β*, have been shown to be partly responsible for the cell proliferation, apoptosis, differentiation, and development [44]. ER-*α* is the principal ER expressed in the endometrium, and it is considered to be crucial in the development of endometrioid endometrial carcinoma [45]. In addition, expression of ERs is elevated in the adenomyotic uteri [46], and ER-*α* is associated with the onset and growth of adenomyosis [47]. Consequently, an assay on the mRNA level of ER-*α* can help us understand the mechanism underlying the effectiveness of SGD in the treatment of adenomyosis. By qRT-PCR analysis, we found that SGD treatment remarkably inhibited the expression of ER-*α* mRNA.

Although the cause for OTR overexpression in adenomyosis is currently unclear, it has been shown that OTR is involved in adenomyosis-associated dysmenorrhea and may be potential therapeutic target in treating the symptom and perhaps chronic pelvic pain in women with adenomyosis [48, 49]. Meanwhile, myometrial OTR expression is also found to be associated with the severity of dysmenorrhea in women with adenomyosis [48]. In the present study, we found that treatment of human adenomyosis-derived cells with SGD could significantly reduce the expression of OTR mRNA, leading, possibly, to pain alleviation. This finding supported the traditional use of SGD in the treatment of adenomyosis-associated dysmenorrhea.

In addition, it is widely accepted that the combined action of multiple constituents in traditional Chinese medicine is considered to be crucial for its therapeutic effects [50]. Yin et al. reported that total 58 compounds from SGD have been detected by UPLC-ESI-Q-TOF-MS, and 50 compounds among them were identified or tentatively characterized including monoterpene glycosides (albiflorin and paeoniflorin), flavonoids (liquiritin, isoliquiritin, and isoliquiritigenin), and triterpene saponins (glycyrrhizic acid) [51]. Meanwhile, UPLC method has been described for the determination of albiflorin and paeoniflorin in rat plasma [52], and it has also been used to simultaneously determine the concentrations of paeoniflorin, liquiritin, and glycyrrhizic acid in the transport samples [53]. Moreover, pharmacokinetic profiles of monoterpene glycosides (albiflorin, oxypaeoniflorin, and paeoniflorin), flavonoids (liquiritin, isoliquiritin, liquiritigenin, isoliquiritigenin, and ononin), and triterpene saponins (glycyrrhizin and glycyrrhetinic acid) in rat after oral administration of SGD extracts by HPLC-MS/MS have been revealed [54, 55]. In the present study, UFLC profiles of two single herbs and SGD were performed for quality control of SGD. A simple and reliable UFLC method was developed for the simultaneous determination of paeoniflorin and liquiritin in SGD. Paeoniflorin is one representative monoterpene glycoside in* Paeoniae Radix*. Liquiritin is a kind of flavonoids that is extracted from* Glycyrrhizae Radix*. Furthermore, in order to reveal the active constituents and their contribution to the effect of SGD, we also examined the antiadenomyosis effects of its 2 major ingredients, paeoniflorin and liquiritin, on human adenomyosis-derived cells. Results showed that both paeoniflorin and liquiritin significantly reduced proliferation, accelerated apoptosis, and suppressed ER-*α* mRNA and OTR mRNA expression in human adenomyosis-derived cells. Paeoniflorin can also decrease production of PGE_2_ and PGF_2*α*_. These findings suggested that the antiadenomyosis effects of SGD on human adenomyosis-derived cells would be partially achieved by these 2 components. In previous studies, it was reported that paeoniflorin possessed analgesic [56], anti-inflammatory [57], immunoregulatory [58], cognition-enhancing [59], neuromuscular-blocking [60, 61], and antihyperglycemic [62] activities, could relax vascular smooth muscle [63], and suppressed rat adjuvants arthritis by reducing COX-2 expression [64]. Liquiritin exhibited an anti-inflammatory effect in some models of inflammation [65], could induce apoptosis in stomach cancer cell [66], and is frequently used to treat injury or swelling for its life-enhancing properties as well as detoxification in traditional Oriental medicine [67]. These evidences may partially support our present findings. Taken together, our findings suggested that paeoniflorin and liquiritin might be bioactive components of SGD. However, further research is needed to evaluate the biological and pharmacological effects of other constituents of SGD.

## 5. Conclusion

In conclusion, our study showed that SGD and its major constituents (paeoniflorin and liquiritin) displayed a differential ability to inhibit proliferation and induce apoptosis of human adenomyosis-derived cells. SGD and paeoniflorin effectively decreased the production of PGE_2_, PGF_2*α*_. Meanwhile, SGD, paeoniflorin, and liquiritin significantly suppressed the expression of ER-*α* mRNA and OTR mRNA in human adenomyosis-derived cells. The results of the present study suggest that SGD and its major constituents (paeoniflorin and liquiritin) possess active antiadenomyosis activities.
